# Leveraging Single-Cell RNA Sequencing Experiments to Model Intratumor Heterogeneity

**DOI:** 10.1200/CCI.18.00074

**Published:** 2018-04-17

**Authors:** Meghan C. Ferrall-Fairbanks, Markus Ball, Eric Padron, Philipp M. Altrock

**Affiliations:** ^1^ **All authors:** Moffitt Cancer Center and Research Institute, Tampa, FL.

## Abstract

**PURPOSE:**

Many cancers can be treated with targeted therapy. Almost inevitably, tumors develop resistance to targeted therapy, either from pre-existence or by evolving new genotypes and traits. Intratumor heterogeneity serves as a reservoir for resistance, which often occurs as a result of the selection of minor cellular subclones. On the level of gene expression, clonal heterogeneity can only be revealed using high-dimensional single-cell methods. We propose using a general diversity index (GDI) to quantify heterogeneity on multiple scales and relate it to disease evolution.

**MATERIALS AND METHODS:**

We focused on individual patient samples that were probed with single-cell RNA (scRNA) sequencing to describe heterogeneity. We developed a pipeline to analyze single-cell data via sample normalization, clustering, and mathematical interpretation using a generalized diversity measure, as well as to exemplify the utility of this platform using single-cell data.

**RESULTS:**

We focused on three sources of patient scRNA sequencing data: two healthy bone marrow (BM) donors, two patients with acute myeloid leukemia—each sampled before and after BM transplantation, four samples of presorted lineages—and six patients with lung carcinoma with multiregion sampling. While healthy/normal samples scored low in diversity overall, GDI further quantified the ways in which these samples differed. Whereas a widely used Shannon diversity index sometimes reveals fewer differences, GDI exhibits differences in the number of potential key drivers or clonal richness. Comparison of pre– and post–BM transplantation acute myeloid leukemia samples did not reveal differences in heterogeneity, although biological differences can exist.

**CONCLUSION:**

GDI can quantify cellular heterogeneity changes across a wide spectrum, even when standard measures, such as the Shannon index, do not. Our approach can be widely applied to quantify heterogeneity across samples and conditions.

## INTRODUCTION

In many cancers, there still exists a critical need to understand the mechanisms of the evolution of therapy resistance. For example, acute myeloid leukemia (AML) is an aggressive hematologic malignancy the hallmark of which is the proliferation of immature myeloid cells in the bone marrow and life-threatening ineffective hematopoiesis.^1^ AML is the most common adult leukemia, with an incidence of approximately 20,000 cases per year and a 5-year survival of only 26%.^2,3^ Diagnosis of AML requires greater than 20% of myeloid immature cells (myeloblasts) in peripheral blood or bone marrow. Median survival of untreated AML is measured in weeks.^4^ Several AML targeted therapies have been recently approved—for example, midostaurin for patients with FLT3 mutated disease and enasidenib for those with mutations in IDH2.^5,6^ These mutations occur at rates of 25% (FLT3) and 5% (IDH2) of all patients with AML and their targeted therapies are generally well tolerated compared with their chemotherapeutic counterparts.^7^ However, midostaurin—and even more potent FLT3 inhibitors in clinical trial^8^—does not fully eradicate disease, which leads to refractory or relapsed AML in most patients.^9^ Complete response rate for enasidenib in relapse/refractory IDH2 mutated AML is less than 20%. Additional refinements in patient selection are required to realize mutationally directed therapy.^5^ Little is known regarding the emerging resistance mechanism and whether targeted therapies—single or combination—against AML alone can ever be successful.

Conventional dogma postulates that therapeutic resistance occurs via the acquisition of mutations that result in clonal evolution. Emerging data suggest that these mutations are either subclonally present or present at frequencies detectable using digital polymerase chain reaction or ultradeep sequencing technologies at diagnosis or before progression. Low-level somatic mutations are also detected in preleukemic states.^10-13^ Somatic mutations are often present years before the diagnosis of therapy-related myeloid neoplasms.^14,15^ Of interest, these mutations are commonly associated with disease progression and transformation.^16^ The presence of such low-frequency genetic markers suggests that high levels of intratumor heterogeneity (ITH) persist over long periods of time and that preexisting ITH is a primary driver of future therapy resistance, whereas variation in transcription over time shapes the disease phenotype. A clinically relevant summary metric by which to describe ITH on the transcriptional level has not been developed.

Single-cell RNA (scRNA) sequencing technologies can present a cost-effective method with which to identify transcriptomic heterogeneity and directly measure ITH. Proof-of-concept studies have been performed in AML using DROP sequencing that yields potentially cost-effective single-cell annotations of thousands of transcripts per cell.^17^ In triple-negative breast cancer, intercellular heterogeneity of gene expression programs within tumors is variable and correlates with genomic clonality.^18^ A study in chronic myeloid leukemia demonstrated that scRNA sequencing was capable of segregating patients with discordant responses to targeted tyrosine kinase inhibitor therapy.^19^ These data provide a rationale by which to explore ITH in scRNA sequencing data and to determine whether defined measures of ITH can be predictive of progression, eventually leveraging this process to mitigate progression and relapse.

The goal of the current study was to quantify ITH in cancer such that it has maximal predictive value, in particular in hematologic malignancies. To this end, we present a platform that uses a generalized diversity index that characterizes cell population heterogeneity across a spectrum of scales (orders of diversity).^20^ These scales range from clonal richness (low order of diversity reveals the number of distinct subpopulations), to more classic measures, such as Shannon or Simpson indices (intermediate order of diversity), to the number of most abundant cell types that can possibly act as key drivers of heterogeneity before transformation or perturbation by therapy (high order of diversity).

## MATERIALS AND METHODS

We created a computational and modeling approach to develop a robust statistical picture of the persistent and emerging variability in scRNA sequencing data chiefly on the basis of on DROP sequencing technologies; the 10× Genomics platform offered a variety of data sets linked to disease and treatment dynamics.^21^ We specifically used the data sets of two healthy/control bone marrow mononuclear cell samples (BMMCs) and two individuals with AML BMMCs sampled pre– and post–bone marrow transplantation (BMT) to develop and test our ITH pipeline.

**FIG 1.**
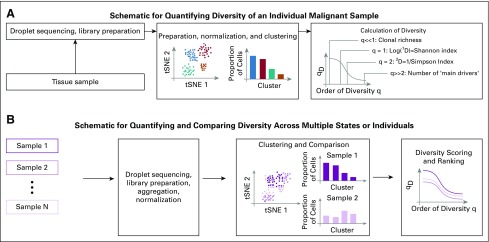
Schematic of our single-cell RNA sequencing–based approach. (A) Workflow for calculating a generalized diversity index for a single sample. After sequencing and library preparation, normalization to reduce the number of false negatives or false positives is applied—for example, using the 10× Genomics platform. Clustering can then be applied (Loupe Cell browser or other platforms; Data Supplement), from which we can calculate diversity. (B) A similar approach can be used when multiple samples are compared. Data normalization and clustering now have to be implemented considering all samples (Data Supplement), and diversity scoring can inform a ranking of intratumor heterogeneity across samples. Single dots in the t-distributed stochastic neighbor embedding (tSNE) plots represent single cells, which might either be marked according to their cluster classification or according to their sample of origin.

**FIG 2.**
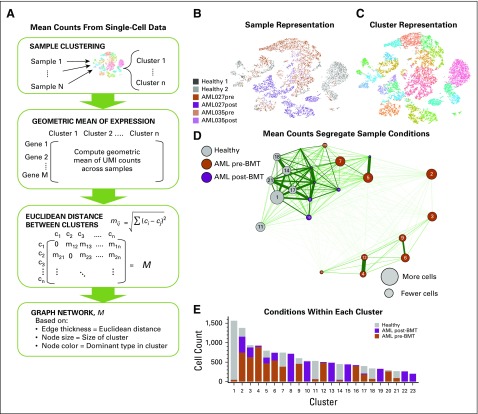
Mean cellular gene expression across clusters within patients can separate disease conditions to some degree. Here, we built a network on the basis of mean differences in overall expression. (A) We calculated the geometric mean of unique molecular identifier (UMI) counts across samples and genes for each cluster. Then, a Euclidean distance was calculated between clusters. Here, we used publicly available single-cell RNA sequencing data^21^: two healthy donor bone marrow mononuclear cell samples (BMMCs) and BMMCs from two patients with acute myeloid leukemia (AML) pre–bone marrow transplant (BMT) and post-BMT. These six samples were then clustered using 10× Genomics Loupe Cell browser (for alternative clustering methods see the Data Supplement), for which we show the (B) sample-based and (C) cluster-based t-distributed stochastic neighbor embedding (tSNE) plots from the Loupe browser. Each dot represents a single cell, which is colored either according to sample of origin or its assigned cluster. (D) Cluster-based differences in mean gene expression over UMI counts gave rise to a clustering of the clusters. Nodes in the resulting graph were colored on the basis of the dominant cell type from each condition present in each cluster (gray for healthy, orange for AML pre-BMT, and purple for AML post-BMT), and the distance between nodes was chosen inversely proportional to the difference in mean gene expression level. (E) Individual distributions of cells from a specific condition in each cluster are shown.

First, we ran publicly available FASTQ-format files—a typical output from a DROP sequencing experiment—through the cellranger count pipeline and then through the Cell Ranger aggr pipeline to pool the samples together for comparison during cluster analysis, interrogated through the 10× Genomics Loupe Cell Browser (Data Supplement). To test the robustness and validity of our diversity metrics and the ITH pipeline, we extended our analysis to include additional publicly available data sets for other hematopoietic cell types (CD34^+^, CD14^+^, CD19^+^, and CD4^+^),^21^ as well as matched normal-tumor lung cancer samples from six patients,^22^ for which we used the same approaches and pipelines. To calculate summary metrics—outlined in Figure 1—first the transcript expression data were clustered into groups of cells with similar transcript expressions (Cell Ranger aggr). We next quantified the distance between each of the clusters to determine if clusters separated on the basis of healthy or disease status (healthy *v* AML). A Euclidean distance was calculated between mean expression values for each gene of each cluster to establish a distance metric (Fig 2).

**FIG 3.**
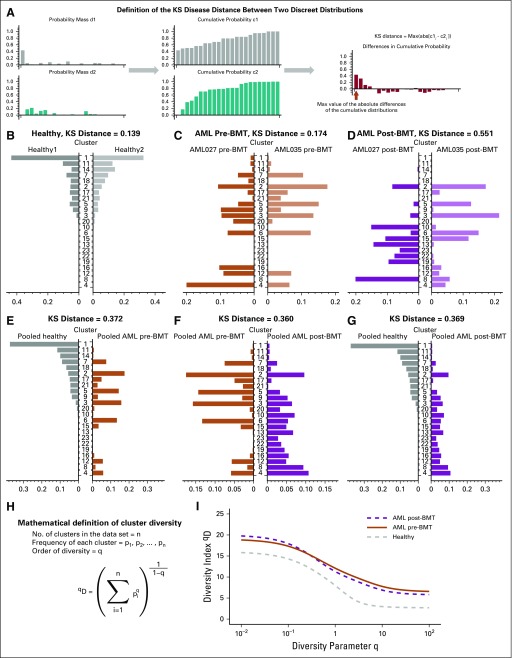
Cluster-based diversity scoring reveals strong differences between healthy individuals and patients with cancer. In our analysis, using data from Zheng et al,^21^ we evaluated our ability to score significant differences in cluster diversity across healthy and acute myeloid leukemia (AML) samples pre– and post–bone marrow transplant (BMT). (A) As a first indicator of between-sample or between-condition differences, we used the Kolmogorov-Smirnov (KS) distance for discrete probability mass functions. (B and C) Little difference was found in the KS distance within condition differences, except in (D) post-BMT samples. (E-G) Between-condition differences were larger when comparing pooled samples across conditions. (H) We calculated a general diversity index (GDI) ^q^D to quantify diversity across orders of diversity q. (I) For all orders of diversity measure, patients with AML (pre- and post-BMT) had a higher diversity index compared with healthy individuals (two samples per condition), which suggests that GDI can be used as a metric for stratification.

Second, we sought to characterize across-sample differences by calculating the Kolmogorov-Smirnov (KS) distance^23^ of the cell count distributions in each cluster. This was done to compare samples or pooled samples of the same condition—for example, disease versus healthy—in terms of the cellular distribution over the identified clusters (Figs 3A-3G).

**FIG 4.**
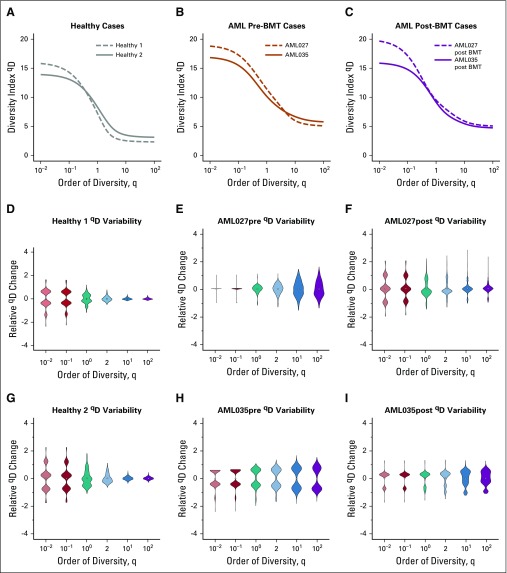
Patients with acute myeloid leukemia (AML) have consistently higher diversity compared with healthy individuals. Individual diversity spectrums were reported for (A) healthy, (B) AML pre–bone marrow transplantation (BMT), and (C) AML post-BMT samples (each line is from one sample). Cell-gene matrices were downsampled to 50% of the cells 1,000 times, and ^q^D scores were calculated using the full pipeline (see Data Supplement) for specific values of q = 0.01, 0.1, 1, 2, 10, and 100. Distributions of relative ^q^D changes for (D and G) healthy, (E and H) AML, and (F and I) post-BMT AML samples showed that, in general, lower q values lead to less change in measured diversity. Across all cases, the diversity score did not change by more than two units (relative change is measured by dividing the entire distribution by the distribution mean). For sample sizes, see the Data Supplement.

Third, we calculated an ecological diversity index^24^ using the cellular frequencies over clusters across a range of order of diversity (Figs 3H and 3I). To assess the robustness of our diversity metric, we performed downsampling of the original data sets and found the relative change in diversity index across a range of order of diversity to determine the sensitivity of our diversity metric (Fig 4).

**FIG 5.**
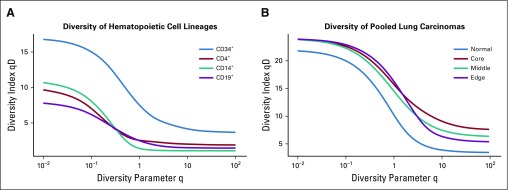
Higher diversity indicates higher clonality in normal tissues and solid tumors. (A) Additional available data from Zheng et al^21^ for CD34^+^ cells, CD4^+^ helper T cells, CD14^+^ monocytes, and CD19^+^ B cells were run through pipeline S (Data Supplement), and the continuum of diversity was calculated for each population. The naturally polyclonal population (CD34^+^) shows the highest diversity score. Each of the other differentiated immune cell compartments are more homogeneous across orders of diversity. (B) In solid tumors, location matters. Normal tumor matched lung carcinoma samples were obtained from publicly available data for six patients with lung cancer^22^ (individual patients; Data Supplement). The diversity metric across q demonstrates an increase in diversity within tumors across different tumor locations.

Last, we applied our ITH pipeline and diversity metric to two additional data sets—a hematopoietic cell-type data set that compared CD34^+^ cells with CD4^+^, CD14^+^, and CD19^+^ cell populations,^21^ as well as a lung cancer data set with matched normal-tumor tissue sites taken from six different patients with lung cancer^22^ (Fig 5 and Data Supplement). Additional specific details of our methods, such as cells per sample, are described in the Data Supplement and are available online (including all code used to generate our results).^25^

## RESULTS AND DISCUSSION

We established a proof of concept that we can generate clinically relevant summary metrics of ITH by analyzing publicly available scRNA sequencing data.^21,22^ Within BMMC samples from diagnosed AML and healthy control groups, we sought to establish how to summarize both inter- and intrasample ITH. First, we clustered the transcript expression of two healthy individuals and two patients with AML, each sampled twice, once before and once after allogenic BMT.

As verification, we sought to distinguish between healthy and AML samples on the basis of the mean expression values across cells and across clusters (Fig 2A). With 23 clusters identified (Figs 2B and 2C), a network of clusters emerged, displayed as an undirected graph where the distance between mean unique molecular identifier counts determines the thickness and length of the edges (Fig 2D). The size of the node was chosen to indicate he total number of cells in the cluster. We colored each node according to the condition—health, pre-BMT AML, or post-BMT AML—that was in the majority (breakdown of actual proportions per cluster shown in Fig 2E). This indicated that the large clusters with mostly healthy cells are most similar in average gene expression, whereas the large clusters with mostly AML cells cluster separately (to the right). Post-BMT cells clustered more closely to the healthy samples than to pre-BMT AML samples. This result supports the idea that these patients were potentially still transitioning but closer to a healthy phenotype; however, some AML-dominant clusters still grouped near the healthy/post-BMT super cluster. On the basis of this bulk measure alone, one may not be able to easily distinguish between healthy and diseased cells. Therefore, other quantifications and metrics to describe gene expression differences may better discriminate between patients with different clinical presentations and staging.

To determine metrics that are better at discriminating between healthy and disease AML, we analyzed and summarized inter- and intraheterogeneity in two different ways. First, we considered the grouping of cells into clusters of similar gene expression in each sample. To this end, we used KS distance, which compares two discrete probability mass functions (the fraction of cells per cluster; Fig 3A). We identified similar distributions within the same condition and different distributions between conditions, with post-BMT being a notable exception (Figs 3B-3D). KS distance between the two healthy samples was 0.139 and 0.174 between the two pre-BMT AML samples, but between the two post-BMT samples it was 0.551. As we had clustered all six samples together, we could also compare them pooled by condition, which revealed that conditions distribute differently across the identified cellular subpopulations in high-dimensional gene expression space (Figs 3E-3G).

Second, we calculated a general diversity index (GDI) for each condition (healthy, pre-BMT AML, and post-BMT AML). The mathematical definition of GDI, ^q^D, is shown in Figure 3H. We established segregation of the different clinical conditions according to this ecology-based diversity index.^24^ Pre-BMT AML samples had a consistently higher diversity index compared with the healthy sample. This held true across the entire order of diversity range, q (Fig 3I). Of interest, on this level, post-BMT samples also scored unanimously higher in GDI. This could indicate that post-BMT settings may require a certain amount of time after transplantation to evolve toward a healthy spectrum of intraleukemic diversity. In addition, in a comparison of the individual samples within each condition (Figs 4A-4C), post-BMT samples were most different from each other.

To interrogate the robustness of GDI further and to establish confidence in the metric, we downsampled the data set, then reclustered and calculated the ^q^D spectrum (Figs 4D-4I). During downsampling, we analyzed each sample individually by randomly removing 50% of the cells, then calculating the number of clusters identified for that individual’s transcript expression, and finally calculating the diversity index for specific q values of interest, including q = 10^−2^; q = 10^−1^; q = 1, which relates to the Shannon index; q = 2, which defines the inverse of the Simpson index; q = 10; and q = 10^2^. Distributions shown were obtained from 1,000 runs of independent downsampling. Intriguingly, these distributions showed that with removing one half of the cells, diversity scores did not change more than one or two units in either direction. Compared with the diversity spectrum shown in Figure 3I, this suggests that if healthy diversity spectra were shifted up by two units (10% of the maximum) and AML samples diversity spectra were shifted down by two units, there would still be visible separation between the healthy and AML conditions.

To further validate our metric, we implemented on our approach with two other data sets. One data set described different hematopoietic cellular subtypes—CD34^+^, CD4^+^, CD14^+^, and CD19^+^.^21^ CD34^+^ is a hematopoietic progenitor cell marker and represents a polyclonal population that includes many different subtypes—hematopoietic stem cells, multipotent progenitor cells, common myeloid progenitor cells, common lymphoid progenitor cells, megakaryocytes erythroid progenitor cells, and granulocytes macrophage progenitor cells—all of which express CD34.^26-28^ The CD34^+^ polyclonal population contrasts with the CD4^+^, CD14^+^, and CD19^+^ populations, which represent more homogenous cellular populations (helper T cells, monocytes, and B cells, respectively). This clonality pattern was recovered by GDI (Fig 5A), where the CD34^+^ population had a considerably higher diversity score across the spectrum. Of interest, lower q values seem to separate differentiated cells more robustly.

Finally, we quantified ITH using lung cancer scRNA sequencing samples.^22^ We analyzed six different patients, each with up to three different tumor sites—core, middle, and edge—and a patient-matched adjacent normal lung tissue sample. Using our GDI metric, we see that the diversity spectrum of the normal lung tissue was much lower than any of the tumor site diversity scores (pooled conditions; Fig 5B). Of interest, more clear separation of conditions was achieved at high orders of diversity q, which indicated differences in the number of driver clones at different sites within the tumors. These additional data sets further support the ability of a quantified diversity metric to discriminate between healthy and diseases states, which can be applied in a clinical setting.

In conclusion, scRNA sequencing efforts have helped greatly to uncover population structures and mapping to specific cellular population patterns.^29^ Although these methods can also elucidate tumorigenesis^30^ and immune profiles,^31^ as well as detect and track genomic profiles of clones,^32,33^ the overall utility of scRNA sequencing for cancer progression survival metrics has been elusive.^34^ Here, we demonstrate the potential utility of two scRNA sequencing–based scores of cellular heterogeneities using a GDI that may be elevated in disease. Remarkably, using previously published data, without additional processing, our quantification of intratumor heterogeneity was able to accurately distinguish AML from healthy individuals as well as from post-transplantation conditions. These data suggest that ITH can be estimated using diversity-based summary statistics and that these summary statistics can be leveraged to predict clinical outcome.

The aim of the current study was to optimize and identify a clinically relevant summary index for ITH in the context of AML, for which targeted single-cell genome sequencing was also able to sensitively uncover complex clonal evolution.^32^ We anticipate that our intraleukemic heterogeneity (ILH) metric will be prognostic for leukemia-free survival and potentially overall survival, even after correcting for known clinical prognostic variables. We have also demonstrated how this metric can also be used to effectively describe heterogeneity in other malignancies, including such solid tumors as lung carcinomas.

From a clinical perspective, in terms of tumor heterogeneity and the emergence of resistance clones during targeted therapy,^35,36^ we expect our metric can discriminate patients clinically. We hypothesize that these heterogeneity metrics would be elevated independently—at least a priori—in potentially highly resistant patients. The advantage of the more general metric used here is that it allows us to look across many orders of diversity and potentially pick a desired range of heterogeneity quantification. For example, one might be interested especially in lower q values, where a higher diversity score may indicate an individual (sample) more at risk for resistance evolution as it shows high standing variation. In contrast, differences at high q values point to key differences in the number of important driver clones, which might uncover distinct vulnerabilities that can be targeted in combination or adaptively.

Diversity measures have long received attention in ecology and evolution.^20,37^ Here, we measured diversity—and thus tumor heterogeneity—using a general definition of nonspatial diversity^38^ in the form of ^q^D quantity (Figs 3H-3I). This approach considers all possible orders of diversity q, but also allows for the comparison of disease stages according to a specific diversity index (fixed choice of q), which emerge as special cases of ^q^D. Species (clonal) richness of a sample is given by q = 0. The Shannon index (log scale) can be found when q approaches 1. The Simpson index, which approximates the probability that any two cells are identical, emerges from the case of q = 2. Both the Shannon and Simpson indexes have been used in mathematical and statistical models of cancer evolutionary dynamics to quantify tumor heterogeneity as it potentially changes during tumor growth, with disease progression, or during treatment.^39-41^ Shannon entropy-based statistics have also been used to quantify single-cell heterogeneity to deliver insights into emerging or disappearing clones during transitions between clinical conditions.^42^

scRNA sequencing experiments provide a snapshot of the cell population on the level of gene expression and can characterize how transcriptomes of individual cells compare with the bulk. In contrast to mass cytometry, DROP sequencing is fast and extremely high throughput. Other single-cell technologies, such as flow cytometry, can be used to generate single-cell data for a relatively small subset of potential markers that distinguish between normal and disease. This requires that the researcher or clinician know what these markers are in advance. Among a variety of outcomes that may be distinguished by our metric, one can be the study for segregating samples on the basis of on disease severity, for which additional follow-up knowledge will be needed.

Further extending our results to the potential impact in a clinical setting in leukemias, ILH is a known reservoir for tumor resistance and clinical refractoriness to targeted therapies.^9^ Clinical responses have been modest to current targeted therapies for the treatment of AML. Specific means to change ILH can be of particular appeal in such cases, as they might help render tumors less aggressive and hinder their ability to rapidly evolve resistance. In particular, hypomethylating agents—cytosine analogs that irreversibly bind to DNA methyltransferase, an enzyme that is required for methylation of CpG-rich DNA—have the potential to diminish ILH.^43^ Transcriptome changes upon treatment with hypomethylating agent therapy have not been analyzed at single-cell resolution. Our analyses in the current study provide a quantitative basis by which to understand and reliably track these changes.

Clear separation of diversity metrics by condition, as we show it, might not be expected in general. A weakness of our approach is that it does not consider any meaning of the associated phenotypes or genotypes; therefore, as it stands, our method cannot be transferred to improve the predictive power of existing bulk signatures. Hence, existing survival data are unlikely to be useful to prove that GDI is predictive of survival, and novel databases that uniquely connect high-throughput single-cell experiments with clinical outcomes are needed.

However, once the appropriate cohorts are established, changes in an individual’s diversity score could indicate unique features of disease progression. In the context of adaptive therapy,^44,45^ which aims at tumor burden control rather than difficult tumor eradication, it might be critical to identify the appropriate scale of diversity that best predicts outcomes. One could speculate that there is an optimal window of diversity that should be maintained—low diversity could indicate fast disease progression and high diversity could mean that the tumor could adapt to the treatment schedule too quickly. The concept we have introduced here is sufficiently flexible in its ability to quantify optimally predictive windows of diversity that should be maintained during adaptive therapy.
